# Maintaining psychological well‐being when living at risk of Huntington's disease: An interpretative phenomenological analysis

**DOI:** 10.1002/jgc4.1965

**Published:** 2024-09-09

**Authors:** Hollie Cooper, Jane Simpson, Maria Dale, Fiona J. R. Eccles

**Affiliations:** ^1^ Division of Health Research Lancaster University Lancaster UK; ^2^ Leicestershire Partnership NHS Trust, Mill Lodge, The Rise Narborough UK

**Keywords:** Huntington's disease, interpretative phenomenological analysis, lived experience, psychological well‐being, qualitative

## Abstract

Living at risk of a genetically inherited disease can be a challenging experience causing psychological distress as well as the possibility of the genetic disease leading to physical health problems. Huntington's disease (HD) is a genetic, neurodegenerative condition. It causes motor dysfunction, cognitive decline and, during the progression of the disease, different psychological difficulties are common. A total of 12 participants living at risk of HD were interviewed and interpretative phenomenological analysis methodology was used to understand their experiences of maintaining psychological well‐being. This resulted in three themes: (1) “you're constantly in limbo”: living in two worlds; (2) “I have to live, just bloody live”: managing the possibility of a time‐limited lifespan; and (3) “I try and try my hardest to look past the disease”: the exhausting quest to keep living well. The findings indicated a need for improved knowledge within professional settings, such as for family doctors, counselors, and other health professionals, specific strategies that genetic counselors can use to support this group, and provision of accessible support and implementation of systemic interventions that would offer support for psychological coping strategies and communication around well‐being to the individual and their family unit. Future research could contribute to the formation of such knowledge and the provision of HD‐aligned services to help support the psychological well‐being of people living at risk of HD.

**What is known about this topic**

Very few studies have been conducted on the experience of managing psychological well‐being while living at risk of HD. From the studies which have touched on this issue, similar levels of psychological distress have been reported for those living at risk and those who have been tested and found to have the genetic expansion for HD.

**What this paper adds to the topic**

This paper illuminates the experience of managing psychological well‐being while living at risk of HD. It presents insights into the complex skills and strategies used by participants to manage psychological well‐being such as living in two worlds and living life as well as possible, as well as the persistent exhaustion created by living at risk of HD, despite the psychological well‐being strategies they formed.

## 1 INTRODUCTION

Genetic diseases often have profound, progressive, and irreversible physical effects on individuals. Where such a condition is not present at birth, but develops later in the person's lifetime, evidence also suggests that even before any physical changes, individuals can experience considerable psychological challenges. Nonetheless, genetic testing, diagnosis and, where available, genetic counseling can help form treatment and management plans to minimize the distress caused by, for example, the potential physical implications of such conditions (Walter & Emery, 2012) and threats to identity (Klitzman, 2009). However, less is known about the wellbeing of those “at risk” individuals, that is, those who choose not to partake in seeking testing or diagnosis despite a high risk of being affected. As they may have minimal contact with health or other services, they have been largely ignored in terms of the effects of their “at risk” status on their wellbeing. An example of one such condition where many at‐risk individuals choose not to pursue genetic testing is Huntington's disease (HD) (Baig et al., 2016).

HD is a genetically inherited neurodegenerative disease caused by a CAG triple repeat expansion in the Huntingtin gene (MacDonald et al., 1993). A recent systematic review of global incidence and prevalence estimated 0.48 cases per 100,000 person‐years for the former and 4.88 per 100,000 people for the latter (Medina et al., 2022). HD symptoms progress over time, for approximately 10–20 years (Ready et al., 2011; Roos et al., 1991), causing progressive motor dysfunction, cognitive decline (leading to dementia), and distress (Heiberg, 2008). The disease is extremely debilitating in the later stages, leaving individuals unable to speak, needing a wheelchair, and experiencing severe involuntary movements (Klein et al., 2014). At present, there is no cure for HD though treatments for some effects of the condition, such as involuntary jerking (chorea), are available (Dash & Mestre, 2020).

As HD is genetic, children of a parent with the expanded gene have a 50% chance of inheriting the condition (Rivera‐Navarro et al., 2015). Offspring who are informed of their 50% risk can experience negative emotions (Folstein et al., 1983) similar to the grief process experienced following a traumatic event (Evers‐Kiebooms & Decruyenaere, 1998). If the genetic expansion is inherited, the person will develop HD, usually between 30 and 50 years of age (Di Maio et al., 1993; Roos et al., 1991) when the person moves from a premanifest (movement symptom free) state to experiencing motor symptoms of the disease.

Individuals are “at risk” of HD when a biological parent (or grandparent when the parent's status is unknown) has HD. Such individuals who are aged 18 and above can, in most countries, request a genetic test to see if they have the HD gene expansion before symptoms occur. However, research indicates that uptake in genetic testing for HD is low, with over 80% of those at risk choosing not to take the test (Baig et al., 2016). Hawkins et al. (2013) argue that individuals who decide to take the test are often met with barriers to testing that are not only practical, for example, regarding finances, but also psychological such as increased distress. HD services are largely inaccessible to individuals who are not engaged in the route to testing and do not have either the symptoms or a diagnosis of HD (Etchegary, 2011) meaning that those living at risk of HD are unable to access specialist support. When non‐specialist services are accessed by those living at risk, healthcare providers' knowledge of HD is reported as being too poor to enable appropriate support (Skirton et al., 2010). This gap in services may mean that genetic counselors are ideally placed to offer support and signposting when they meet with at‐risk people either through another family member seeking testing, or via those who access genetics but decide not to pursue testing.

Research into the psychological needs of adults who have not pursued testing is limited. For example, the Prospective Huntington At risk Observational Study (PHAROS) in the US (Quaid et al., 2017) reported that people living at risk carried a significant burden around disclosure and concealment of their at‐risk knowledge. While further analysis from this study highlighted some of the day‐to‐day experiences of living at risk (Quaid et al., 2008), it did not offer understanding of the psychological or emotional experience outside of the study's themes focused on risk (disclosure or concealment) and living in hope of being negative. Further quantitative research (Chisholm et al., 2013) found that HD has a complex impact on wellbeing. Alongside other findings, the study suggested no significant difference in psychological wellbeing between those at risk and those who had pursued genetic testing. The study suggests that greater focus is required to understand the impact of the risk of HD on well‐being. While previous research offers us some insight into the potential impact of HD on well‐being, existing studies are focused on the impact at specific points in the lifespan. For example, Sparbel et al. (2008) focused on teenagers' (14–18) experiences of growing up with the knowledge that HD was within their family. Similarly, Forrest Keenan et al. (2007) explored the experience of young people (between 9 and 28 years of age) finding out about HD in the family, understanding their own risk and the impact of HD on their relationships. Aubeeluck et al. (2011) also added to the understanding of the impact of HD in their study focused on carers. These studies inform us that HD has a significant impact on people at various stages and roles of life. While they offer some insight into the mechanisms and strategies that might be used to manage this impact, this is not explicitly stated and it remains unclear whether such strategies and mechanisms are used throughout the life span of those living at risk. For example, while Etchegary (2009), in this study's comparison of those living at risk, those who tested positive for the pathogenic variant and those clinically affected by HD, identified three main modes of coping, the emphasis was very much on coping and not the management of psychological well‐being.

Consequently, the current study explored the experience of maintaining psychological well‐being for adults living at risk of HD, including the coping mechanisms and strategies used. Such knowledge can inform appropriate healthcare provision for this group, which is currently minimal (British Psychological Society, 2021; Zarotti et al., 2020). The research question to guide this exploration was: What is the experience of maintaining psychological well‐being for those living at risk of HD?

## 2 METHOD

### 2.1 Methodology

Interpretative phenomenological analysis (IPA) was used as a qualitative method with an idiographic focus that allows the consideration of individual lived experiences in informing meaning (Smith et al., 2021). IPA explores human experience (phenomenology) and how such experience is understood (hermeneutics) and was originally designed to understand the experiences of people with chronic health conditions (Smith et al., 1999). The theoretical underpinnings of IPA (phenomenology, hermeneutics, idiography) recognize that experience needs to be examined closely in research, that is, initially at the individual level, as opposed to a more broad or distant approach. The flexibility offered through the use of IPA, in its focus on lived experience and interpretation, presents an in‐depth method of meaning‐making concerning the experience of maintaining psychological well‐being when living at risk of HD.

### 2.2 Participants

Participants were recruited via two pathways within which a digital poster was shared with contact details for those interested in taking part. The first was via an online HD social media group, the second via the social media account of an HD charity, both UK based. The inclusion criteria ensured that the purposive and homogeneous sample required for IPA was achieved (Smith et al., 1999). Smith et al. (2021) suggest that an ideal number of participants for IPA is between four and ten individuals as smaller samples allow for deeper understanding to be achieved. To allow for potential withdrawal and to ensure the gathering of sufficient data, 18 participants were recruited from 112 initial expressions of interest in order of response time. Six participants did not attend interviews or respond to follow‐up contact. This resulted in 12 completed interviews (four via the HD social media group and eight via the HD charity). Nine participants were female, with three male. Participants were aged 19–66 years. All participants were white British.

To be included in the research, participants were required to be aged 18 and over, aware of their at‐risk status and able to communicate where their risk originated allowing level of risk (25% via grandparent, 50% via parent) to be established. Guidance suggests individuals need around 6 months for adjustment to this knowledge, and a further 6 months for the experience of living with this knowledge (Tibben et al., 1997). Therefore, participants needed to have knowledge of their at‐risk status for a minimum of 12 months. In line with the inclusion criteria, participants self‐reported as symptom free, not tested for HD, spoke English without any communication difficulties and were able to participate in an interview (or two if needed) of up to 90 min in length. An excerpt of the topic guide can be seen in Table 1. Participation was voluntary and participants could withdraw at any point up to 2 weeks after the completion of their interview. At this point data analysis had begun, meaning extracting individual data would be difficult. Demographics of the participants are presented in Table 2. All names are pseudonyms to preserve anonymity.

**TABLE 1:** Examples of the topics in the topic guide.

Who in your family has been diagnosed with Huntington's?
What is your understanding of living at risk?
How do you keep yourself psychologically well?
What do you do to cope with the knowledge of HD?
Does living at risk impact how you care for yourself?
Do you think living at risk impacts your wellbeing?
What are your experiences of wellbeing?
Have you ever sought support for psychological distress? If yes, what was this like?
Have you ever sought support to explore living at risk?

**TABLE 2:** Participant demographic details.

Pseudonym	Age	Gender	Relationships status	Biological children	Employment status	Family member with HD	Length of time aware of HD risk	Likelihood to have the expansion (%)	Accessed psychological support
Sarah	43	Female	Married	2	Full time	Father Grandmother Great Uncle	Long known history of HD – unsure of when knowledge of risk was gained	50	Y
Jane	60	Female	Widowed	0	Self‐employed	Father Grandmother Aunt Uncle	Long known history of HD – unsure of when knowledge of risk was gained	50	Y
Lucy	37	Female	Married	1	Full time	Mother	Long known history of HD – unsure of when knowledge of risk was gained	50	Y
Marie	66	Female	Married	0	Retired	Sister Mother x2 Uncle Grandfather	Long known history of HD – unsure of when knowledge of risk was gained	50	N
Daniel	45	Male	Long term relationship	0	Full time	Mother Sister	9 years	50	N
Lisa	33	Female	Married	1	Full time	Mother Grandfather	13 years	50	Y
Ian	24	Male	Long term relationship	0	Full time	Father	8 years	50	Y
Gill	53	Female	Married	1	Self‐employed	Mother	18 years	50	Y
Karen	19	Female	Single	0	Unemployed	Father	12 years known of HD, understood risk later though unsure when	50	Y
James	43	Male	Married	1	Full time	x2 brothers cousin	14 years	50	Y
Cath	40	Female	Married	0	Full time	Mother	32 years known of HD Understood risk later though unsure when	50	Y
Evelyn	23	Female	Single	0	Full time	Grandfather	4 years	25	Y

### 2.3 Procedures

Ethical approval to conduct this study was given by the first author's academic institution (FHMREC20188). *All applicable international, national, and/or institutional guidelines were followed in the conduct of the study*. Participants were given an explanation of the study via a participant information sheet and a consent information sheet explaining audio consent. *All participants then gave informed consent, verbally replying to the consent questions, before the main interview*. Participants were interviewed using Microsoft Teams due to COVID‐19 restrictions and to ensure the sample was not limited by location in the UK. Interviews were recorded and transcribed verbatim by the first author.

### 2.4 Data collection

Data were collected using semi‐structured individual interviews conducted by the first author in December 2021 and January 2022. Interviews lasted for approximately 1 h. The semi‐structured interview schedule was developed using existing literature for guidance on creating topic guides (Busetto et al., 2020; Murray & Wilde, 2020; Pietkiewicz & Smith, 2014) and topic guides shared within existing qualitative HD research (Quinn et al., 2010; Stopford et al., 2020). The topic guide was finalized with experts‐by‐experience. This helped establish areas for focus (such as experiences of distress, the causes and how these were managed, strategies people used to stay psychologically well, how people made sense of HD and risk in their own lives), exploration and the phrasing of questions in a curious and explorative way suited to a phenomenological approach (Smith et al., 2021).

### 2.5 Data analysis

Participants' narratives were transcribed verbatim with identifying details (e.g., places, names) removed. The most recent IPA guidelines were followed for analysis, creation of themes, and use of terminology (Smith et al., 2021). The analysis involved the idiographic approach of reading each transcript individually and following the seven steps described by Smith et al. (2021). The first of these involved reading and re‐reading the transcripts to enter the participants' world and during this process, the author was required to keep an open mind (Smith et al., 2021). The second involved making exploratory notes, noting phrases of interest or possible significance. The third involved the formation of Experiential Statements, that is, statements which were directly related to the participants' experience. The fourth involved the identification of similarities and connections across the Experiential Statements. This allowed the Experiential Statements to be grouped to form Personal Experiential Themes (PETs). In the fifth stage, the PETs were named and placed in a table. Step six involved repeating this process (steps one to five) for each participant until each participant had a table of PETs. Once this was completed, the final step involved analyzing PETs across participants and grouping those which were similar to form Group Experiential Themes (GETs). At this stage researcher interpretation is integral to the formation of themes.

The primary author generated the GETs and the interpretation of these themes. As this is an intersubjective dynamic, reflexivity (Finlay & Gough, 2008) was addressed through the keeping of a reflexive journal so the author could remain aware of the lens through which data were being explored. The primary author had no lived experience that aligned with participants in this research; therefore, it was important to be aware that social and cultural beliefs may have affected her perceptions of people living at risk of HD. Furthermore, to ensure the findings were rooted in the participant data and to improve the trustworthiness of interpretations, data analysis was supported by the three co‐authors through a discussion of understanding participants' experiences throughout the IPA process. This ensured that the four benchmarks of quality in qualitative research – sensitivity to context, transparency, coherence, and importance (Yardley, 2000) were also considered.

## 3 FINDINGS

Through analyzing the transcripts, three themes were generated: (1) “you're constantly in limbo”: living in two worlds; (2) “I have to live, just bloody live”: managing the possibility of a time‐limited lifespan; and (3) “I try and try my hardest to look past the disease”: the exhausting quest to keep living well. Where quotations have been shortened, this is indicated with a bracketed ellipsis (…).

### 3.1 “You're constantly in limbo”: Living in two worlds

This theme explores how participants managed psychological well‐being by moving between HD and non‐HD‐dominated worlds. Living in the HD world involved an awareness of being at risk and of the experiences around HD that were constantly present and causing distress. Living in the non‐HD world explored the respite and relief participants experienced when they moved out of the HD world.

Some participants ensured they had a group of friends or an environment in which HD was invisible. These participants had genuine, invested relationships with separate groups of friends (those who knew about HD and those who did not) but would choose with whom to socialize depending upon how they were feeling about their risk. Being able to spend time with those with no knowledge of HD offered participants an identity independent of HD and a period of respite from having to engage consciously with the condition. For some participants, this social separation seemed to enable a recharging of resources. For example, for Ian, it was important to be able to keep HD out of his working life but have the flexibility to speak to people he chose to about HD:

…it's still a secret, but it's a controlled secret now, it's not, it's not taken over my life…. (Ian)

Similarly, other participants described how they were able to wear a “mask” that eliminated HD when they wanted to be in the non‐HD world. The “mask” was used in work, within social circles or for their children and family. This gave a feeling of accomplishment and control, that is, that they could silence HD when they needed to and be fully present as themselves, making a choice to remove the “mask” when they were ready to enter the HD world.

As well as choosing when to access the non‐HD world, participants could also choose to dwell in the HD world. Some participants' experiences suggested that choosing to spend time in the HD world had a beneficial outcome amid their distress. Spending time with people who knew about HD provided a shared experience that offered a sense of belonging, acceptance and understanding that brought connection and a release:

…it just feels good inside knowing that other people [know about HD], so that's why at the same time seeing more people and talking to more people helps as well as like, as a coping mechanism as opposed to just keeping it locked up inside, cause that ain't good for no‐one, that. (Ian)

Being able to discuss and explore HD openly with people who understood provided an outlet for stress and worry and relief for participants as they did not have to embark on the tiresome repetition of explaining their relationship with HD.

However, as well as being able to choose to live in one world or the other, participants could be forced from one to the other. This resulted in higher levels of distress and participants fought to regain control. For example, to control distress triggered by worries around the onset of symptoms, which would force them into the HD world, participants described tests they would do. The aim of these tests was to evidence that the onset of HD had not occurred. This reassurance enabled participants to reduce distress and move between HD worlds again. For some, this testing process would be conducted multiple times throughout the day. Some participants described tests as cognitive tasks such as puzzles, reciting names, places, and details of songs whereas other participants might use a physical test:

I have a habit of when I'm in bed, of rubbing my feet together and I, like, try and hold, stop doing it to see how long I can hold it, so (….) I can really discover whether they're involuntary or not. (Lisa)

Participants also used perspective to control the pull into the hopelessness of the HD world by comparing their lives to the lives of others. This brought short‐lived relief that they were “*lucky (…) it's (HD) nowhere near as bad*” (Marie). This could be in comparison to tragedy reported on the news or, more specifically, other people experiencing HD symptoms:

…reading other people's (HD) stories and I think it makes me, in a really horrible way, makes me feel better about my own situation because, you see younger people like suffering, oh my gosh I'm stressing about something I might get when I'm 60. (Lucy)

However, this was temporary and the realization that this did not change their situation pushed them back into the HD world and the overwhelming awareness of the *“disgusting disease” (Lisa)*.

….trying to think it could be so much worse but it's, it's quite difficult to maintain that, I think you think it for a few minutes and “yeah, you're lucky it could be worse”, and then it sort of hits you like a sack of shit afterwards that you're still at risk. (Lisa)

Ultimately it seemed that being able to step into the non‐HD world brought temporary respite and a period of recuperation before the awareness of their at‐risk status and their HD‐related life experiences hit them again. For all participants, time was required to be spent in both worlds to maintain a sense of well‐being and balance.

### 3.2 “I have to live, just bloody live”: Managing the possibility of a time‐limited lifespan

This theme describes the experience of living in an HD‐free period and the awareness that being HD‐free may end. It explores the pressures of a possible time‐limited life span, the need to be worthy of an HD‐free life, and the battle to gain control over the onset of symptoms. While this pressure had many negatives, there appeared to be positive factors involved with the idea of living within a pre‐determined lifespan.

Participants spoke of the pressure to live their lives within an allocated window of HD‐free time that was largely based on their family narrative concerning the age of onset. For some this was the idea of a non‐HD life until their 70s due to previous family members' age of onset and, for others, this was non‐HD life until their 30s:

…all the time, every day it is a constant, ‘oh well, I'm losing time’ I know, I know everyone's got an expiry, but with this it feels like it's just that's it, personally I thought for the past seven years that as soon as I hit thirty I'm gone. (Ian)

For those whose family members started with symptoms during their working life, a psychological goal was set that seemed to offer a sense of safety, achievement and relief. For example, some participants spoke of how, if they “made it to retirement age”, they would feel they had escaped HD as they had been able to experience all aspects of the lifespan. Interestingly this timespan seemed to be linked to the period of life (working) rather than chronological age.

Participants strived to live a “*full life*” (Cath) before HD, with an intense sense of pressure to fit in as much as they could. Participants seemed to use this pressure to give them energy and motivation to live life to the full. For example, creating a list of goals or accomplishments to be achieved prior to symptom onset (for example, buying a house, having children, learning to drive) was presented as a “*motivating force to keep going*” (Cath). However, it seemed that the experience of doing this in such a time‐pressured way removed some of the joy of the experience and the celebration of the achievement. There was no time to celebrate and reflect on the achievement as the next goal was waiting.

For most, despite their efforts to live a full life, the threat of the future arrival of HD stole long‐term goals and dreams. For example, one participant shared how their dream to relocate to a rural location had been abandoned due to the possible need to access health care. Others described how they felt that life was constantly making them aware of the possible impact of HD on their future when they witnessed their in‐laws enjoying their retirement, grandchildren, or traveling.

…my mother and father‐in‐law go on such amazing holidays now they're retired and I just kind of think, you can't take that for granted…they have such a nice life…they go on cruises and play with the grandchildren, and it does upset me that there's a 50% chance that might not happen for me. (Lucy)

This led to an intense sadness, grief, envy, and sometimes anger that they might “*not get the chance*” (Sarah).

Some participants explained that they had a fear that HD would cause their ability to empathize and be kind to people to deteriorate. To control the level of deterioration experienced, participants spoke of starting at a higher point:

…and I think I made a decision that I would be really nice to everybody, all the time, because then, if it got me as I'd have, if my starting place was at really one end of the spectrum when I was really such a lovely, nice person, then perhaps I'd die before I became evil, which is magical thinking isn't it? (Jane)

The idea of being able to start at a higher point or having a scale of effort was continually referred to when participants spoke of ways they tried to limit the impact of onset should they have the genetic expansion. Knowing that such effort had been made seemed to bring a sense of control over onset but also a prediction of peace if onset did begin: “*…I'm just trying to get everything done as soon I can…..I realize I can slow down a bit now*” (Ian). Some participants aimed to care for the body and mind as well as they could through diet, exercise and tasks designed to keep the brain active:

…the longer I could keep my brain active, and you know, keep going, I think the healthier connections will stay and the longer my neurology can, yeah, I can keep exercising my brain for as long as possible, hopefully it will protect itself a little bit. I mean that's wishful thinking, there's no evidence that will happen, but I think for me that what I want and, so, yeah, I can't let myself shut my brain down and like stop thinking. I've got to try and keep, keep going. (Sarah)

Participants knew there was no evidence to suggest such techniques had an impact on onset, though this did not remove the importance of active efforts to feel like onset was in their control. By doing this hard work, participants were able to reduce their anxiety with the self‐reassurance such behaviors brought. If they developed symptoms, they would be more peaceful in their suffering knowing they had done everything in their power to prevent onset and lived a good life for as long as they could.

Ultimately, it seemed even when participants aimed to live life to the full, HD stole from their experiences with added pressure, a sense of having to rush and the acknowledgment that they could still lose life experiences related to old age. No matter how fast they lived, this area of life remained out of reach.

### 3.3 “I try and try my hardest to look past the disease”: The exhausting quest to keep living well

Within this theme, the participants' experience of being perpetually exhausted by living at risk is explored. Exhaustion came from many areas, such as the worries about HD taking their physical body, the challenge of finding their own identity independent of HD, family‐related stressors and inconsistent access to information and poor support from professionals.

Participants spoke about the difficulties they experienced in being able to keep HD in the “*back of their head*” (Sarah) so they could be themselves and how unexpected observations, such as seeing a particular gait of an individual, would move HD to the front of their mind.

It's hard. I do think about it most days, not dark, not in a dark way, I just it's there and you know, I mean it's there and I know it's there and sometimes when I'm in the street and I see somebody they might be struggling and I could tell it's neurological, I think maybe they've got Huntington's disease and I often think ‘I hope you got some support. I hope you're not on your own’. (James)

It seemed that, for participants, trying to find their own identity outside of HD was an exhausting task overshadowed by fear of losing oneself. Losing the mind and the parts of themselves that made them who they were and not being able to do the activities they enjoyed, as well as being met with societal judgment, were the greatest fears expressed about developing HD.

…we've had issues when we've gone out shopping with Mum before where people have been really rude and abusive to her because they assume that she's drunk. (Daniel)

…in a few years' time I might not be able to sit down and read books like my Dad, that was one of the first skills that he lost, the ability to concentrate on reading a book and that thought of not being able to read again terrifies me more than having the physical symptoms…. (Sarah)

The biological manifestation of the disease and its progression was difficult though manageable but the loss of self was described as “*terrifying*” (Lisa). This fear seemed to be driven by the idea of losing their identity, the activities they loved (for example being able to read, revisit memories, laugh), and experiences with unhelpful cultural narratives. Narratives that called HD the “*devil's disease*” (Lisa) and those who experienced it “*devil's spawn*” (Jane) or “*drunk*” (Daniel) were intensely distressing and affected self‐perception:

How can I be loveable when it's called the devil's disease? (Jane)

Participants who experienced public shaming of their relatives were deeply hurt. Such experiences resulted in a tiring psychological process of preparing for social outings followed by a needed recovery period to process the stigma encountered.

I mean, my Mum looks likes she's been on the lash [drunk] constantly. (Gill)

It was apparent such distressing social interactions took away the small victories participants tried to implement for their symptomatic family members, leading to a sense of hopelessness:

…that's the kind of situations you face sometimes, when you're trying to do the best for your family who are suffering, and you're trying to make them have as much joy as what you have, and then I guess they just get put in a corner sometimes. (James)

Another source of exhaustion could be seen when participants described thinking about their family history and relationships. It seemed important to participants to distinguish between the person and the disease and recall the positive traits and memories of the person prior to HD and retain them throughout their HD deterioration. This often brought a circular questioning which resulted in a “*Pandora's box*” (Gill) of questions concerning whether they were parented by a person or a disease:

…I've never really known him without symptoms, but in my head, I try and sort of picture what he was like, and kind of look at the photos and like tell myself that it was HD that made him do those things, and not, it wasn't my Dad kind of thing…. (Karen)

Feelings of helplessness experienced by participants seemed to drain their ability to remain in control of their distress. As participants were forced to watch their family members experience years of difficulties, it seemed as though they were unable to provide relief for their loved ones or their own suffering for any lengthy period. This experience would often feed back into their own worries about the possibility they were witnessing their own future.

..you know in the back of your mind that's this is gonna be me or my sister one day….he's our Dad but also knowing that it's going to be probably our future as well. (Karen)

This triggered the need for more strategies to be put into action to cope, using more of their energy resources.

All participants described challenging interactions with health professionals which made living at risk more difficult, stressful and tiring. This seemed to add to their levels of exhaustion and reinforced feelings of being alone, disconnected and unseen:

..I speak to my therapist. She's not specifically trained in Huntington's disease (…) I don't think she understands it very much (….). She said that she doesn't think that my mum's got Huntington's and she said that she thinks that I know deep down that I don't have Huntington's (….). Saying stuff like that isn't helpful, because, I'm trying to come to terms with the fact that I am at risk (….) and she's saying things like, well, I don't think you have it. (Evelyn)

Ultimately, when reflecting on experiences with professionals, all participants spoke of how they felt unheard and questioned “*where are our voices?*” (Jane). This resulted in feelings of isolation and exclusion that led to intense sadness, low mood, anger, and loss of hope that their situation could change, and an unwanted idea, presented by professionals and current health care systems, that whatever they did, success was unlikely.

## 4 DISCUSSION

This analysis of individuals' experiences of maintaining psychological well‐being while living at risk of HD resulted in three themes. The themes suggest that maintaining psychological well‐being was a complex challenge that required the implementation of multiple strategies.

In the first theme, *“you're constantly in limbo”: living in two worlds*, participants spoke about the movement between the HD and non‐HD world. This is similar to experiences reported by people affected by HD in Sweden (including individuals at risk, negative, and pre‐symptomatic, and members of the family affected by HD) (Hagen, 2018) and Canada (at risk or a family member not themselves at risk) (Etchegary, 2009). Such findings are in line with earlier research which has concluded that living with HD is a fluid and dynamic experience and awareness of risk has a fluctuating prominence over life for those living at risk and their families (Cox & McKellin, 1999).

Participants needed specific skills to make movement between the HD and non‐HD world consistently available to them. Participants spoke of the importance of having separate groups of friends who might or might not know about HD or periods of time where they did not need to think about HD in their current conversation or environment. Participants had a varied age range and length of knowing about HD and risk, though this did not seem to impact their ability to move between the two worlds. Other factors seemed to be influential such as having a reliable social support network and being able to control one's own thoughts about their risk. Having a positive mindset and understanding risk seemed to have a more significant impact than age or length of knowing. For all participants, this movement was not an attempt to deny the existence of HD in their lives but a provision of respite from the distress the awareness of the disease brought and an opportunity to be themselves independent of HD. Pakenham et al.'s (2004) study of stress and coping in HD compared at‐risk individuals who had decided to take the genetic test versus those who had not and suggested that those who did not had a greater reliance on passive, avoidant coping. However, here it is clear that the situation is more complex for non‐test takers as rather than avoiding HD the participants moved between the two worlds, sometimes engaging with the HD world and sometimes not, in order to manage their well‐being. Furthermore, Pakenham et al. actually found similar levels of social support and locus of control between the two groups and indeed for both groups found these variables, along with others such as control appraisals and were both predictive of adjustment. The current study also highlights the importance of social support and a sense of control. Importantly Pakenham et al. also found although they differed on some coping variables, the two groups had similar levels of adjustment and suggested other factors such as optimism may be important; as noted above, a positive mindset was important here. Participants carefully managed which friends should and should not know about their risk. Making decisions about with whom to share their HD risk is a technique found to be effective in other HD research to assist coping (Quaid et al., 2008). In Quaid's study, conducted in the USA, the importance of concealment of at‐risk status was a key theme and decision making about disclosure was impacted by the expected reactions of friends and colleagues, but also concerns around health insurance and discrimination. Worries about reactions from future partners when dating were also present. This contrasts with the current study in the UK, where the main concern was being able to have places and friends where there was relief from thinking about HD. There were worries about future stigma if they should develop symptoms, but less about the stigma of currently being at risk. Instead, participants moved between HD worlds as they decided which world they were capable of interacting with when they were emotionally stronger and had the resources to dwell within that particular place.

The second theme, *“I have to live, just bloody live”: managing the possibility of a time‐limited lifespan*, presents an awareness of time. Similar concerns about the salience of time and making the most of the time when healthy are seen in those at the pre‐manifest stage (e.g., Gong et al., 2016; Wieringa et al., 2022). Other chronic illness research also suggests that individuals who know they will develop a chronic illness experience time in a different way from those who do not have such susceptibility (Jowsey, 2016). Participants often changed their plans which enabled them to prioritize their commitments and keep a focus on gaining as much enjoyment and achievement from life as they could. Participants identified the turmoil their attempts to control time caused and the effects of this time pressure and resulting distress on moving between the two HD worlds and the relevant HD identities. Understanding such difficulty is also supported by longitudinal grounded theory work on chronic illness (Charmaz, 1990, 1991), the results of which suggest that the difficulty people face is the balance between the efforts to control time while preserving their self‐identity. All participants expressed an awareness of time and time pressure to live well that seemed independent of their biological age and family narrative concerning the age of onset; participants who were older (e.g., Jane and Marie) did not view their risk as diminishing as they aged, and it seemed their window of time moved with them. This is contrary to the findings by Etchegary (2009) where once the member living at risk had passed their family's average age of onset, this enabled coping.

Socioemotional Selectivity Theory (SST; Carstensen, 2021) seeks to explore the concept of time on life‐span development and suggests that endings, whatever type (e.g., aging, relocating, illness), trigger motivational change. As a result of this motivational change, the person replaces existing goals with more emotionally meaningful goals. The theory has been explored in the context of breast cancer and concluded that SST can provide a useful way to understand motivation and psychological adjustment in people experiencing a time‐limited future due to illness (Sullivan‐Singh et al., 2015). The different factors with the population in this study remain the at‐risk status and the desire to not engage with information in terms of a definitive answer from a genetic test that will place a putative end date in participants' thinking. Without such an end in mind, theoretically this should eliminate the time parameters within which they live; however, this is not the case for participants in this study. Participants in this study did not experience a belief in an expanded lifespan. Their at‐risk status seemed to limit the lifespan in the same way as in other future‐limiting diseases, and indeed in the same way as pre‐manifest individuals.

Theme three, *“I try and try my hardest to look past the disease”: the exhausting quest to keep living well*, explored the tiring work involved in maintaining a sense of well‐being while living at risk of HD. The stress for young people caring for symptomatic family members while thinking about their own risk has been previously documented (Keenan et al., 2007; Sparbel et al., 2008) as have the stresses of caring for a relative with HD more generally, including a lack of professional knowledge and support (Parekh et al., 2018). In the current study with adults of a range of ages, the management of similar stressors, alongside worries of developing symptoms and losing their physical and mental selves, needed considerable work, which was exhausting. The Illness Trajectory Framework (Corbin & Strauss, 1991), focuses on the work of people with chronic illness that is unseen by professionals (Star, 1995) and presents three reciprocally interactive types of work: biographical, everyday life, and illness‐related work. For the current participants illness‐related work included taking physical care of themselves in terms of diet, exercise, and activity and biological work was evident as participants aimed to form an identity independent of HD. The term “everyday life work” could be used to describe usual life commitments such as caring for their family (externally focused) and managing distress (internally focused). The intensity of this work did not diminish as age progressed and participants did not view their risk reducing as they aged. All participants hoped to reach retirement and have considerable time to enjoy that experience. Only then did it seem that peace would be theirs and the hard work could stop, that is, once all areas of the life cycle had been lived.

### 4.1 Study limitations

There are several potential limitations in the current study. IPA relies on the interpretations of the researcher and, while transparency can be evidenced (e.g., through the sharing of transcripts, procedures, and involvement of co‐authors), recognition of the subjectivity of the researcher's interpretations is important. Secondly, participants were recruited via HD‐specific groups on social media platforms. While these groups were not support groups per se, participants spoke of a sense of belonging and understanding gained from being part of such groups. It is possible that recruiting via such groups could have led to selection bias. People involved in such groups may be more proactive in maintaining their psychological well‐being than those living at risk who do not embark in such involvement. It may also be possible that people not involved in such groups have found alternative ways to manage their psychological well‐being and therefore do not require the sense of belonging that these groups offer. This perspective is absent from this study. All participants identified as white British; therefore, future research focused on other groups of participants may be useful to explore a more diverse representation.

Thirdly, the participants in this research were living at risk in the UK, therefore, their experiences of their own cultural context and interactions with the NHS healthcare system need to be considered. Cultural and social context and experiences of accessing medical care or support can influence individuals' perception of illness, such as HD (Arran et al., 2014), which influences the emotional response and coping strategies used to manage illness (Leventhal et al., 1980). Such influences need to be considered when exploring the transferability of the findings to other people living at risk.

### 4.2 Research recommendations

Studies evaluating the effectiveness of psychological interventions for people affected by HD remain limited (British Psychological Society, 2021; Zarotti et al., 2020), therefore research is required to develop and evaluate the use of approaches for those living at risk of HD. The issues raised in this study highlight, as well as the unmet individual needs of this group, the wider systemic issues concerning the impact of social stigma, and lack of knowledgeable professionals and services.

Improving the knowledge of professionals concerning HD would need to be targeted specifically using specific clinical resources such as continued professional development courses or clinical updates. There is also the consideration that such campaigns and educational approaches will not alter the current lack of service provision for those living at risk of HD. While the theoretical models presented may provide a starting point by presenting ways for professionals to increase their knowledge of the impact of living at risk of HD, without further research, funding and implementation, no effective change will emerge for this group.

### 4.3 Practice implications

The current study highlights several issues that can inform genetic counseling practice, in addition to wider healthcare provision. When living at risk, our findings show that the level of intensity required from participants to cope with their risk did not appear to diminish as they aged. This highlights the need for support that is available throughout the lifespan for those living at risk of HD. Furthermore, some of the concerns of the participants, for example, living a limited lifespan, with a pressure to live well, are similar to pre‐manifest individuals (e.g., Gong et al., 2016; Wieringa et al., 2022). This suggests that some of the concerns and therefore support needs may be similar. Given genetic counselors have existing knowledge around these issues for those who test positive, advice and support on managing psychological well‐being could be offered to family members at risk when opportunities arise.

Provisional research has found that psychoeducation in the form of forums was well received by people who were pre‐symptomatic (Gluyas et al., 2023). Consequently, it might be useful to explore whether such an intervention would be useful for the at‐risk population. Moreover, previous interventions involving genetic counseling services and clinical psychologists have been successfully implemented for those who have received either positive or negative genetic test results, and could be adapted to help support people who decide not to pursue testing (MacLeod et al., 2018).

The findings also indicate that there was a need for effective emotional regulation skills (see also Zarotti et al., 2018). Emotional regulation is the ability to experience, make sense of and modulate emotions (Gross, 1998); this is a useful skill in terms of the illness trajectory framework and the level of work needed to stay well. More generally, the ability to self‐regulate when experiencing a chronic health condition is important for adaptive adjustment to illness (de Ridder et al., 2008).

Self‐management techniques were also evident throughout all themes. In chronic illness literature (Dunbar‐Jacob & Mortimer‐Stephens, 2001; Riemsma et al., 2004), good self‐management (e.g., regarding diet, exercise, and medication compliance) has been consistently evidenced as having positive impacts on physical health, due to the care taken to provide wellness for the physical body. The findings in this study highlight participants' engagement in self‐management techniques before any advice, testing or diagnosis is even given. There is a need for professionals to support such explorations and implementation of interventions and strategies.

Participants explored their varied experiences of accessing psychological support. Professionals' knowledge (e.g., that of psychologists, psychiatrists, and therapists) of HD was important to participants, with poor professional knowledge having a negative impact on participants. This experience of HD families was reflected in a recent Italian paper which highlighted that greater HD training was needed among healthcare professionals, and that several barriers existed to getting psychological support for HD (Zarotti et al., 2024). Although specialist HD service provision is sparse, it may be beneficial for existing HD services to employ suitably knowledgeable therapists/psychologists who have knowledge of HD to provide specialist psychological support for those at risk as well as those with HD (British Psychological Society, 2021; Zarotti et al., 2022). Furthermore, greater links between genetic counseling and psychological services could help improve care pathways for those at risk.

Given participants' experience of stigma, awareness campaigns could help raise knowledge and reduce such negative interactions. It has been suggested that making society aware of the negative and harmful impact of such stigma on people with genetic diseases, such as HD, could reduce its occurrence (Edge, 2010; Wexler, 2010). It is important that campaigns that focus on raising awareness are delivered effectively and with clarity and that they have substantial funding provision to create accessible and attractive resources, as insufficiencies in such areas have been shown to make such campaigns ineffective (Wakefield et al., 2010).

### 4.4 Conclusion

This IPA study explored the experience of 12 participants who were striving to maintain their psychological well‐being while living at risk of HD. Participants spoke about the complex day‐to‐day navigation between HD and non‐HD worlds, the drive to live the best life they could in the time they had and the exhausting impact of living at risk of HD. The findings highlight the need for improved support for people living at risk of HD throughout the lifespan and theoretical models that may support this need have been discussed. These include the Socioemotional Selectivity Theory (Carstensen, 2021) to understand the concept of time on lifespan and the ways in which endings trigger change and the Illness Trajectory Framework (Corbin & Strauss, 1991), to explore the work individuals with chronic illness undertake in their everyday life. However, changes to healthcare systems that currently exclude those in need either by the diagnostic eligibility criterion and/or lack of HD knowledge in health settings are urgently needed.

## AUTHOR CONTRIBUTIONS

Authors Hollie Cooper, Jane Simpson, Maria Dale, and Fiona J. R. Eccles contributed to this work. Hollie Cooper made substantial contributions to the acquisition, analysis, and interpretation of data for the study which was supported by Jane Simpson, Maria Dale and Fiona J. R. Eccles. All authors made substantial contributions to drafting the work and revising it critically for important intellectual content. Authors Hollie Cooper and Fiona J. R. Eccles confirm that they had full access to all the data in the study and take responsibility for the integrity of the data and the accuracy of the data analysis. All of the authors gave final approval of this version to be published and agreed to be accountable for all aspects of the work in ensuring that questions related to the accuracy or integrity of any part of the work are appropriately investigated and resolved.

## CONFLICT OF INTEREST STATEMENT

Authors Hollie Cooper, Jane Simpson, Maria Dale, and Fiona Eccles declare that they have no conflict of interest.

## ETHICS STATEMENT

Human studies consent: Ethical approval was granted by Lancaster University Faculty of Health and Medicine Research Committee.

Animal studies: No non‐human animal studies were carried out by the authors of this article.

## Data Availability

The authors elect not to share data.
